# Biodegradation of Vulcanized SBR: A Comparison between *Bacillus subtilis*, *Pseudomonas aeruginosa* and *Streptomyces* sp

**DOI:** 10.1038/s41598-019-55530-y

**Published:** 2019-12-17

**Authors:** Mostafa Galal Aboelkheir, Priscilla Braga Bedor, Selma Gomes Leite, Kaushik Pal, Romildo Dias Toledo Filho, Fernando Gomes de Souza

**Affiliations:** 1 Laboratório de Biopolímeros e Sensores - Instituto de Macromoléculas, Universidade Federal do Rio de Janeiro, Rio de Janeiro, 21941-598 RJ Brazil; 2 Programa de Engenharia Civil, Universidade São Judas Tadeu, São Paulo, 03166-000, Brazil; 3 Escola de Química, Universidade Federal do Rio de Janeiro, Rio de Janeiro, 21941-909 RJ Brazil; 4 Department of Nanotechnology, Bharath University, Tamil Nadu, 600073, India; 5 Programa de Engenharia Civil, COPPE, Centro de Tecnologia-Cidade Universitária, Av. Horacio Macedo, 2030, Bloco I. Universidade Federal do Rio de Janeiro, Rio de Janeiro, Zip code 21941-450, Brazil

**Keywords:** Environmental impact, Characterization and analytical techniques

## Abstract

Rubber residues present harmful impacts on health and environment, besides wasting valuable and huge amounts of rubber. Biological recycling technique is focused here to minimize this problem. A comparison of the biodegradation effect caused by Bacillus subtilis, Pseudomonas aeruginosa, and Streptomyces sp., separately, on vulcanized SBR-rubber during 4 weeks is reported. The surface and molecular analyses were studied by FTIR-ATR, TGA, DSC, TC and SEM/EDS, in addition to the contact angle and crosslinking tests. B. subtilis, P. aeruginosa, and Streptomyces sp. evoked after 4 weeks a loss in v-SBR crosslinks by 17.15, 10.68 and 43.39% and also in the contact angle with water by 14.10, 12.86 and 15.71%, respectively., if compared to Control samples. FTIR findings indicate that the polymeric chain has been partially consumed causing C-C bonds scission indicating the biodegradation and bio-devulcanization phenomena. The bacterial strains caused a carbon loss by 9.15, 5.97 and 4.55% after one week and 16.09, 16.79 and 18.13% after four weeks for B. subtilis, P. aeruginosa, and Streptomyces sp. mediums, respectively. DSC and EDS results are also promising and highlighting Streptomyces sp. strain as the most effective biodegradative one as an alternative and natural mean of degrading vulcanized rubber residues.

## Introduction

Rubber residue represents a significant amount of solid waste as millions of tons of waste tires are generated yearly and discarded after using them, which need a very long time to undergo natural degradation due to the rubber cross-linked structure and the presence of stabilizers and other additives^1,2^. Serious environmental problems appear if the tires are burned in the landfills due to the migration of chemical substances to soil and underground water, leading to the contamination of living organisms, in addition to the air pollution through Persistent Organic Pollutants (POPs) caused by this process if carried out in an uncontrolled big scale^3,4^. Moreover, enormous rubber quantities that come from non-renewable sources are wasted^5–8^.

Worn-out tires once again present a risk for human health and lives as deadly arbovirus vectors (insects that cause diseases like Zika, Dengue, and Chikungunya) take the standing raining water into a discarded tire as a breeding ground. Authorities in many countries, like Brazil and EU, are working on finding innovative means of tire recovery, moreover, on facing illegal disposal in the landfills^9,10^.

To minimize this problem, rubber recycling techniques including Heat Generation via combustion and pyrolysis, chemical/biological recovery and physical recovery, are applied^11–14^. The recovered rubber residue then can be reutilized by its insertion as fillers or additives to polymeric or ceramic matrices allowing new applications^2,15–20^.

Many microorganisms such as bacteria and fungi have biodegradative effects on both natural and synthetic polymers, and many types of research are performed to better understand the mechanisms of the involved biochemical processes^21–23^. The devulcanization process of rubber involves the breaking of the multiple C-S bonds along with the polymeric chains, where removing sulfur makes the recycling of rubber easier. Aerobic and anaerobic microorganisms are being intensively studied as devulcanization prompters, where the metabolic pathways and the enzymes involved, are becoming better known^24–26^. The presence of actinomycetes to metabolize purified natural rubber as the only source of carbon has already been described since 1995, when Heisey and Papadatos, based on fatty acid profiles and cell walls showing seven strains of *Streptomyces*, two of *Amycolatopsis* and one of *Nocardia*, the authors further concluded that actinomycetes were the major organisms involved in the biodegradation of natural rubber hydrocarbons^27^.

There are two groups of bacterial strains that utilize rubber as the only source of carbon and energy based on their biodegradation mechanisms^28–30^. The first group forms translucent halos indicating the excretion of rubber-cleaving enzymes. Between them, Actinomycetes from *Actinoplanes*, *Streptomyces*, and *Micromonospora* are highlighted for their potent results^31^. On the other hand, the second group does not form halos and grow adhesively at the surface of rubber particles in liquid culture, and they represent the most potent rubber-degrading bacterial strains. This group includes *Actinobacteria* belonging to the genera *Gordonia*, *Mycobacterium*, and *Nocardia*. All rubber-degrading species described so far are mesophilic species, with only one exception, identified as a *Streptomyces* sp.^6,32^.

Due to the filamentous nature of actinobacteria growth, a significant shift in molecular weight distribution to lower values could possibly be detected in natural rubber, as reported by Nanthini and Sudesh (2017) using *Streptomyces* sp. strain CFMR7 after 9 months of culture^33^.

According to Yao *et al*., 2013, the microorganisms could break the cross-linked sulfur bonds on the surface of waste latex rubber, however, the main chains remained intact. With the increase of desulfurization time with *Alicyclobacillus* sp., the swelling value of desulfurized waste latex rubber (DWLR) increased, and the cross-link density decreased^34^.

Hu *et al*., 2016, reported that the microbial treatment of vulcanized polyisoprene (v-IR) and vulcanized poly (butadiene-co-styrene) v-SBR showed that *Amicalisa* can break C=C and S-S bonds and the cross-link densities were decreased by 13.7% and 22.1%, respectively, resulting in flexible network portions on the rubber surface^35^.

Other bacterial strains such as *Bacillus* sp., *Nocardia* sp., *Moraxella*, *Pseudomonas citronellolis*, *Gordonia polyisoprenivorans* VH2, and Y2K, G. *westfalica*Kb1 and *Mycobacterium fortuitum* NF4 were also isolated and their biodegradation activity was reported^36–45^.

*Streptomyces* and *Bacillus* are organisms that are commonly isolated from environments such as soils containing a wide range of chemical compounds and have been cited as biodegradable potentials of rubber derivatives^6,33,42^. *Pseudomonas* is also a natural inhabitant of several environments and has been related to the degradation of different organic compounds due to their recognized adaptability in various substrates and metabolic diversity^46–49^. Therefore, these bacteria groups were highlighted as good examples of degrading bacteria to test the activity of each on a single sample applying the same test conditions.

This work is an extensive range study that englobed more than one type of Bacteria in laboratory tests under very controlled conditions using three bacterial strains, which certainly highlights the strain that can be applied on a big scale. By performing a comprehensive range scanning about the different types of bacteria that may offer positive modifications to the rubber residues, the application of such technology can be managed more softly. Also, the synergy effect of a binary strain system, including the most effective strain highlighted in this paper as the leading participant, is intended to be studied. The combination of bacteria strains may lower the cost and the elapsed time of bacteria isolation step.

This paper reports the biodegradation and bio-devulcanization effect caused by B. subtilis, P. aeruginosa, and Streptomyces sp., separately, on a well-known sample of vulcanized styrene butadiene rubber (v-SBR), synthesized at our laboratory. A series of predetermined test conditions was applied as an attempt to standardize the same conditions of treatment and post-treatment characterizations to all the studied samples. A comparison between the three bacteria was set based on surface and/or chemical modifications that occur in the rubber samples leading to the crosslinking declivity. This comparison is supported by applying instrumental analyses exploring the rubber surfaces. To study the synergy effect of this kind of bacteria, future investigations are intended to be performed by our group to test the effect of a binary strain of the most degradable ones.

## Experimental

### Materials

The tested material, Butadiene-Styrene Rubber (SBR)/(Mooney viscosity 51.2 ML 1 + 4 (100 °C), was vulcanized in our laboratory and then was used for this essay. This elastomer was used as an indicative reference for the tire crumb due to the tire complexity as it is made of different rubber types besides SBR, which is heavily applied in tires fabrication^50^. Microorganisms cultivated in Laboratory of Industrial Microbiology (LAMIND-UFRJ) were used in this investigation. The samples were divided into 4 categories: Control, *Bacillus subtilis* ATCC 6633 INCQS/FIOCRUZ, *Pseudomonas aeruginosa* ATCC 9027 INCQS/FIOCRUZ *and Streptomyces* sp., and each has the numbers from 1 to 4 as a function of contact time in weeks between SBR and the medium containing each bacterium type, separately.

### Methods

#### Vulcanization of SBR

The vulcanized SBR (v-SBR) was prepared as recommended by the standards ASTM D 3182 and ASTM 2084. A quantity of 500 grams (g) of SBR was mixed via a roller mixer (KraussMaffei Berstorff-Germany) at 50 °C with zinc oxide, stearic acid as activators, sulfur as a vulcanizing agent and Tetramethylthiuram Disulfide (TMTD) as a vulcanization accelerator. The resulting vulcanized rubber was then crushed in a mill after being immersed in liquid nitrogen.

#### Biodegradation

The nutrient broth consists of meat extract and meat peptone was prepared, inoculated with bacterial strains for 72 hours at 30 °C and then centrifuged via Boeco centrifuge model C-28 A (Germany) at 6000 rpm for 15 minutes. The bacterial suspensions were washed with sterile saline solution 0.9% w/w twice, resuspend and then 1 mL was collected and used as inoculum for the biodegradation assays using the sterile liquid carbon-free basal medium (LCFBM) prepared with 0.7 g of KH_2_PO_4_, 0.7 g K_2_HPO_4_, 0.7 g of MgSO_4_.7H_2_O, 1.0 g of NH_4_NO_3_, 0.005 g of NaCl, 0.002 g of FeSO_4_.7H_2_O, 0.002 g ZnSO_4_.7H_2_O, 0.001 g of MnSO_4_.H_2_O, and 1liter of distilled water, according to Yang *et al*., 2014^51^. Three sets of bacteria strains plus the corresponding nutritive medium were placed into 50 mL transparent glass flasks with 3.75 g of powdered v-SBR, obtained by a cryogenic method using liquid nitrogen and a mill. The rubber particles were classified in different sizes by using different sieves of several openings. The particles that passed through the 300 µm and blocked at the 150 µm sieves were selected to perform this essay. The average particle size of the rubber samples was between 150–300 µm. An equal amount of v-SBR was submitted to a similar nutritive medium without any bacterial strain as the control sample. The test was applied and monitored during 4 weeks at 30 °C and 120 rpm shaker, where each bacterial strain was tested twice a week. In general, a total of 32 samples were under investigation, representing 8 samples of each set: Control, *B*. *subtilis*, *P*. *aeruginos*, *and Streptomyces* sp. After that, all the samples were washed for three hours at 80 °C by distilled water in a mechanical stirring system.

#### Characterization

Scanning Electron Microscopy (SEM): Hitachi TM3000 coupled to Energy Dispersive Spectroscopy (EDS) were used to analyze the morphological modifications of v-SBR, applying acceleration voltage of 15 kV.

Contact Angle with water: The Contact Angle was measured via Data Physics contact angle goniometer model SCA20. Measurements were carried out with water on v-SBR samples at room temperature. The powder SBR was compacted to form a uniform horizontal layer of the microparticles to simulate a solid rigid surface of the same rubber. The needle of the syringe was replaced by a thinner one to produce adequate water droplet not to disturb the compacted rubber surface. Each sample was tested at least three times to check the accuracy of the result.

Chemical group analysis (FTIR): A pastille with 1 mg of each sample and 300 mg of potassium bromide (KBr) was prepared and tested two times via Varian FTIR Excalibur Series spectrophotometer model 3100 (Japan) at room temperature, where a resolution of 4 cm^−1^, 64 accumulated scans and a range from 4000 to 600 cm^−1^ were applied in the transmittance mode.

Thermal Analysis (TGA/DSC): The thermal behavior, thermogravimetric (TGA) and differential scanning calorimetry (DSC) analyses, of the samples, was investigated two times by Perkin Elmer STA 6000. The derivatives of TGA curves (DTG) were applied and smoothed using the software of the same equipment. A heating rate of 20 °C/min from 30 to 700 °C and nitrogen atmosphere with a gas flow rate of 20 mL/min were applied.

Crosslinking Degree: All the measurements were performed applying the leaching method developed in our lab, as described previously^52^. 20 mg of v-SBR were submitted in a tightly closed filter paper to a leaching system for 48 h at the boiling temperature of toluene. The crosslinking degree, related to the insoluble residue, was calculated as the ratio between the weights of the sample after and before being soaked in toluene during the leaching process. Each sample was tested three times.

Mass Balance of Carbon and Sulfur: An amount of 0.2 g of v-SBR was placed into the elemental analyzer (LECO SC144, United States) kiln at 1000 °C to decompose the sample after one and four weeks in the presence of oxygen, and then the percentage of carbon and sulfur are automatically estimated based on the trapped CO_2_ and SO_2_ gases, respectively.

## Results and Discussions

### SEM/EDS analyses

**Figure 1:**
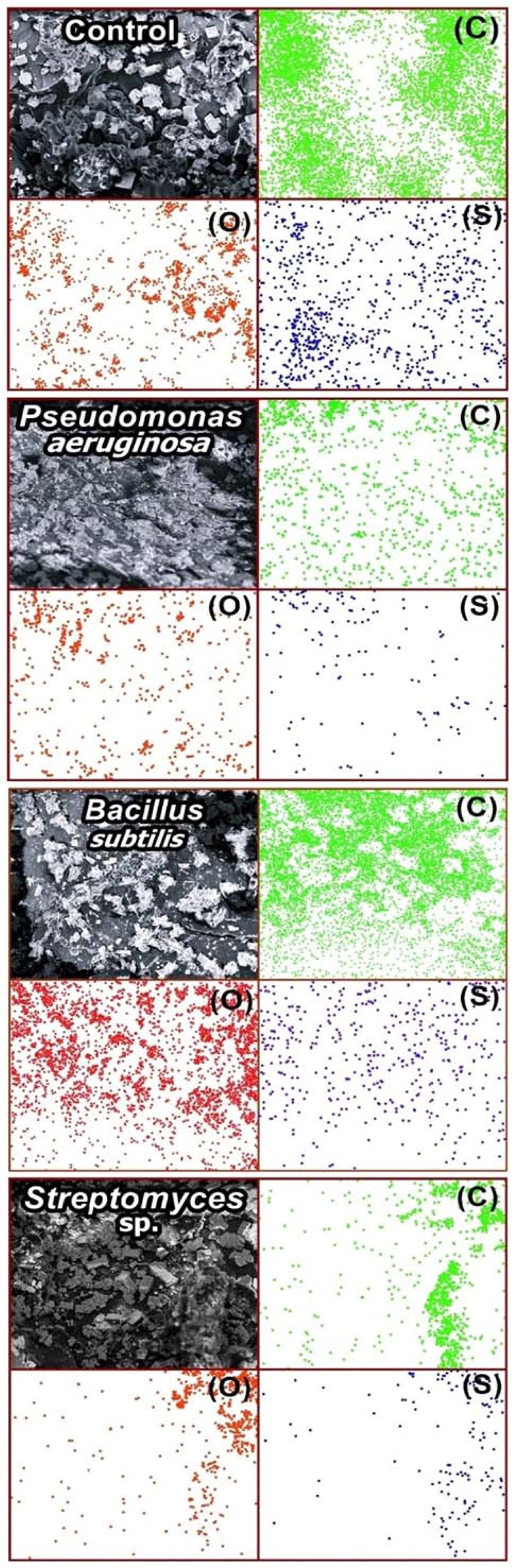
SEM images and the corresponding EDS maps for carbon (C), oxygen (O) and sulfur (S) of the samples Control, *Bacillus subtilis*, *Pseudomonas aeruginosa and Streptomyces* sp.

The three bacteria tend to break the S-bonds on the system leading to the element sulfur being released onto the rubber surface. Thus, sulfur or sulfur compound should exist in lower concentrations compared to the Control samples due to the aggressive washing procedure, described in the methods section. Based on EDS data, sulfur to carbon ratios for the Control, *B*. *subtilis*, *P*. *aeruginosa*, *and Streptomyces* sp. samples are 1.94, 0.47, 0.64 and 0.45%. Table 1 in the SI file shows the EDS Index of carbon and sulfur elemental analysis in v-SBR before and after the contact with three different types of bacteria. All the SEM images of v-SBR rubber before and after the contact with the bacteria at 600x of magnification (100 µm) are shown in Fig. 1. All the micrographs contain brighter points referring to some minerals that were used in the nutritive medium for the bacteria and it could not be easy to remove all of them even by washing using a stirring system. The energy dispersive spectroscopy maps (EDS) of carbon (C), oxygen (O) and sulfur (S) of each sample are presented beside its micrograph in the same image.

### Contact angle

**Figure 2:**
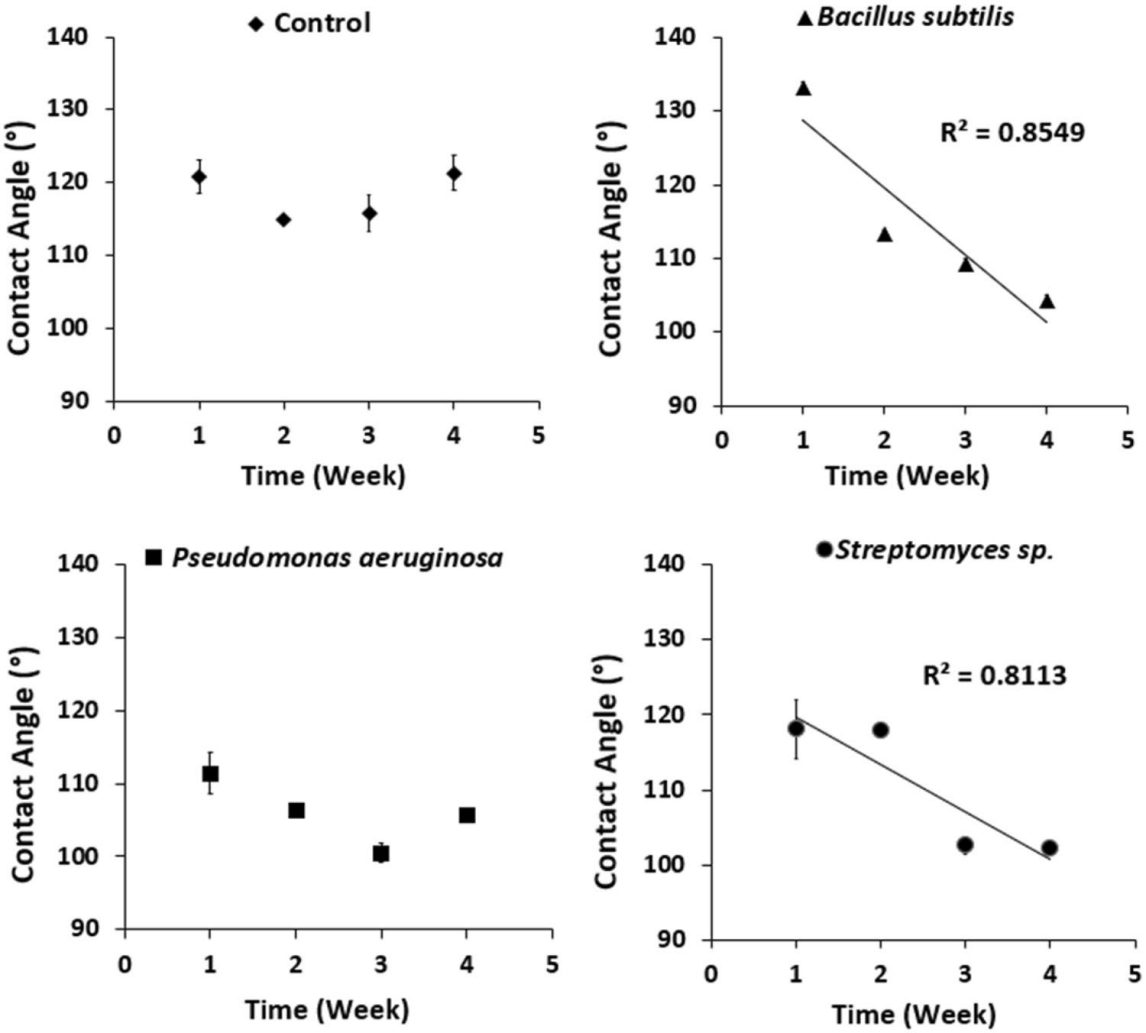
Contact angles of v-SBR rubber modified by different mediums: (**a**) Control, (**b**) *Bacillus subtilis*, (**c**) *Pseudomonas aeruginosa* and (**d**) *Streptomyces* sp. during 4 weeks of contact with the bacterial strains.

**Figure 3:**
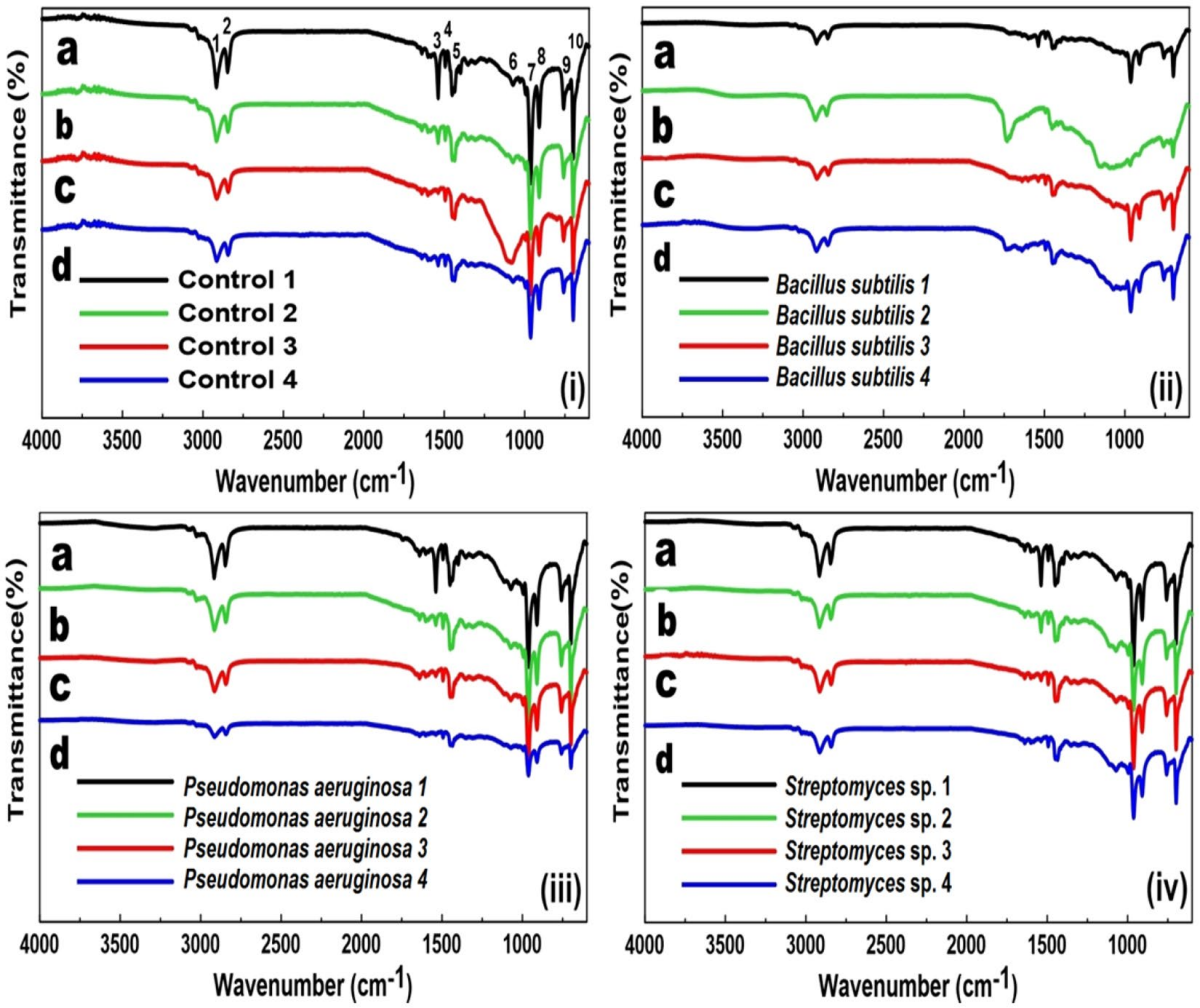
FTIR absorption bands of v-SBR modified by different mediums: (i) Control, (ii) *Bacillus subtilis*, (iii) *Pseudomonas aeruginosa* and (iv) *Streptomyces* sp. during 4 weeks of contact with the bacterial strains.

Figure 2 shows the contact angle graph using water as a solvent. The results are promising and showed that the angles have been decreased by increasing the treatment time indicating higher affinity with water and enhancement in wettability properties for all the rubber samples after the contact with all the bacteria, separately. The Control samples have no significant changes in the contact angles during the studied period; meanwhile, the bacterial strains caused a declination of the contact angles in v-SBR samples, *B*. *subtilis*, *P*. *aeruginosa*, *and Streptomyces* sp., after 4 weeks when compared to the Control samples by 14.10, 12.86 and 15.71%, respectively. A linear regression modeling was applied in the case of *Bacillus subtilis and Streptomyces* sp. (see Fig. 2), where the corresponding correlation coefficient (R^2^) registered 0.855 and 0.811, respectively. This may be due to the biodegradation activity of the bacteria, allowing the rubber surface to be more hydrophilic as polarity tends to increase after possible oxidation of the degraded surface, as shown in FTIR results section (see Fig. 3).

### FTIR analysis

FTIR spectra of the samples are shown in Fig. 3 as follows: Fig. 3-i–iv, presents the v-SBR samples after the immersion during 4 weeks (1, 2, 3, 4) with the Control, *B*. *subtilis*, *P*. *aeruginosa*, *and Streptomyces* sp. mediums, respectively. To avoid discussing the influence of the nutritive solution on the results of this test, the FTIR spectra of the Control sample are considered the comparison basis to detect whether changes had happened or not to v-SBR in the bacterial medium. The bands were named from 1 to 10 to make their identification easier during the discussion.

From Fig. 3, it can be observed that the samples were modified during the contact time between rubbers and bacteria, 1, 2, 3 and 4 weeks. Regarding the v-SBR spectrum, presented in Fig. 3-i, it is possible to observe a doublet at 2916 and 2848 cm^−1^, characteristic bands 1 and 2, related to the stretching of C-H bond in CH_2_ and CH_3_ groups. The C=O stretching characteristic band at 1735 cm^−1^, which is associated with the stearic acid used in the SBR vulcanization process, does not appear in Control samples. The characteristic bands 3, 4 and 5, at 1537, 1493 and 1455 cm^−1^, respectively, are attributed to asymmetric and symmetric stretching of C=C in the aromatic ring skeleton. At 1085 cm^−1^, the characteristic band 6, assigned as S=O and SO_2_ conjugated stretching, appears very weak except for the sample Control 3 (see Fig. 3-ic). Aboelkheir et. al. also reported a very strong and wideband in the same region for non-modified v-SBR in which it has no contact with any solutions^52^. The styrene, cis-1,4-unit, 1,2-unit, and trans-1,4-unit, characteristic bands 7 and 10, show up at 963 and 722 cm^−1^, respectively^53–55^. At 901 cm^−1^, the characteristic band 8, assigned as C-H out-of-plane bend and at 808 cm^−1^, the characteristic band 9, assigned as ring out-of-plane C-H bending^56^.

The characteristic bands 1 and 2 have been slightly reduced in *B*. *subtilis* and *P*. *aeruginosa* (see Fig. 3-ii,iii) while no change was noticed in case of *Streptomyces* sp. (see Fig. 3-iv). The C=O stretching band at 1735 cm^−1^ appeared only in the case of *B*. *subtilis* 2 and *B*. *subtilis* 4 (see Fig. 3-iib,d). This band is weak due to the low concentration of the stearic acid used in the preparation for the vulcanization. Also, a reduction is noticed in the bands 3, 4, and 5 for the bacteria-modified v-SBR samples indicating that was able to cleavage the C=C in the aromatic ring skeleton. All the bacteria-modified samples showed up very weak or almost disappearing bands at 1085 cm^−1^, the characteristic band 6 assigned as S=O and SO_2_, i.e., the S=O and SO_2_ are present in fewer quantities in the modified samples indicating that sulfur bonds were reduced due to the presence of Bacteria. This result corroborates the SEM/EDS findings. This may also indicate a bio-devulcanization of the rubber by causing C-S bonds scission. Moreover, the characteristic bands 7 and 10 registered a new reduction in *B*. *subtilis* and *P*. *aeruginosa* mediums when compared to Control samples (see Fig. 3-ii–iii), but the samples in *Streptomyces* sp. medium maintained very similar features as of Control samples (see Fig. 3-iv). Finally, no changes were noticed for the bands 8 and 9 for the bacteria-modified samples when compared to Control samples. All the vibration modes of the FTIR spectra of v-SBR samples are represented in Table 2 in the SI file. The decrease of the transmittance intensity of the characteristic bands named from 1 to 10 caused by the different types of bacteria indicates that part of the polymeric chain has been consumed causing C-C bonds scission, as well. Thus, the bio-devulcanization process is accompanied by a degradation phenomenon of the rubber. The detected modifications of the polar groups on the rubber surface justify the decrease of the contact angle findings (see Fig. 2).

### Thermal analysis (TGA/DSC)

**Figure 4:**
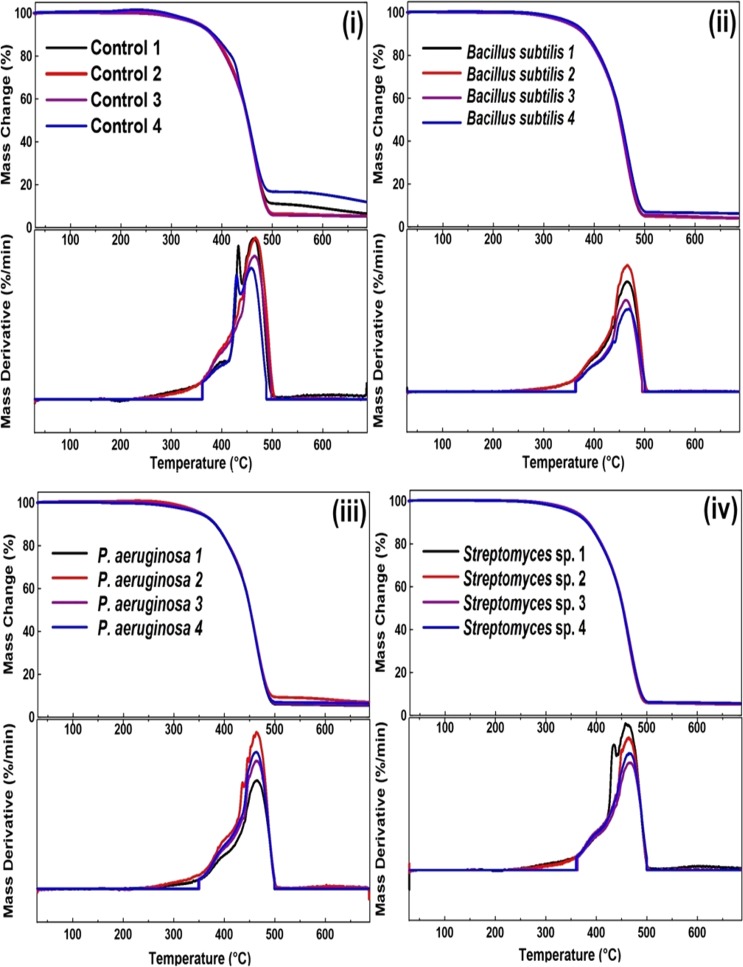
TG/DTG curves of v-SBR modified by different mediums: (i) Control, (ii) *Bacillus subtilis*, (iii) *Pseudomonas aeruginosa* and (iv) *Streptomyces* sp. during 4 weeks of contact with the bacterial strains.

Figure 4 shows the thermogravimetric curves (TGA) and the corresponding derivatives (DTG) as follows: Fig. 4-i–iv, presents the v-SBR samples after the immersion during 4 weeks (1, 2, 3, 4) with the Control, *B*. *subtilis*, *P*. *aeruginosa*, *and Streptomyces* sp. mediums, respectively. Regardless the applied medium with v-SBR, all the corresponding TGA curves are very similar, while slight differences in residual substances due to some inorganic substances, took part in SBR vulcanization process at 700 °C, were observed. Presented TGA results indicate some differences in the structure of the studied v-SBR, which was meanwhile confirmed by the FTIR analysis (see Fig. 3). Cross-link density has a significant influence on thermal stability and thermal behavior of rubber compounds^57^. According to the DTG curves in Fig. 4, for v-SBR two consecutive mass loss events had occurred separately in some cases (two peaks) representing the decomposition temperatures of styrene (peak 1) and butadiene (peak 2)^58,59^.

Table 3 in the SI file shows the degradation temperature after 2, 5, 10, 50 and 85% of decomposition of v-SBR for all the samples after 1 and 4 weeks, as well as, the inorganic residue at 700 °C. Based on data from Table 3 in the SI file, it was observed that there is an overall increasing tendency of T_−2%_, T_−5%_, T_−10%_ T_−50%_, T_−85%_ temperatures, which corresponded with 2, 5, 10, 50 and 85% mass loss, of all the studied v-SBR samples after the fourth week when compared to the first week. The same observation applies in case of the inorganic residue. We reported in our recently published article a very similar case of v-SBR devulcanization by different types of bacteria where we roughly concluded that these tendencies are attributed to the order of breaking the bond presents in the system^52^.

**Figure 5:**
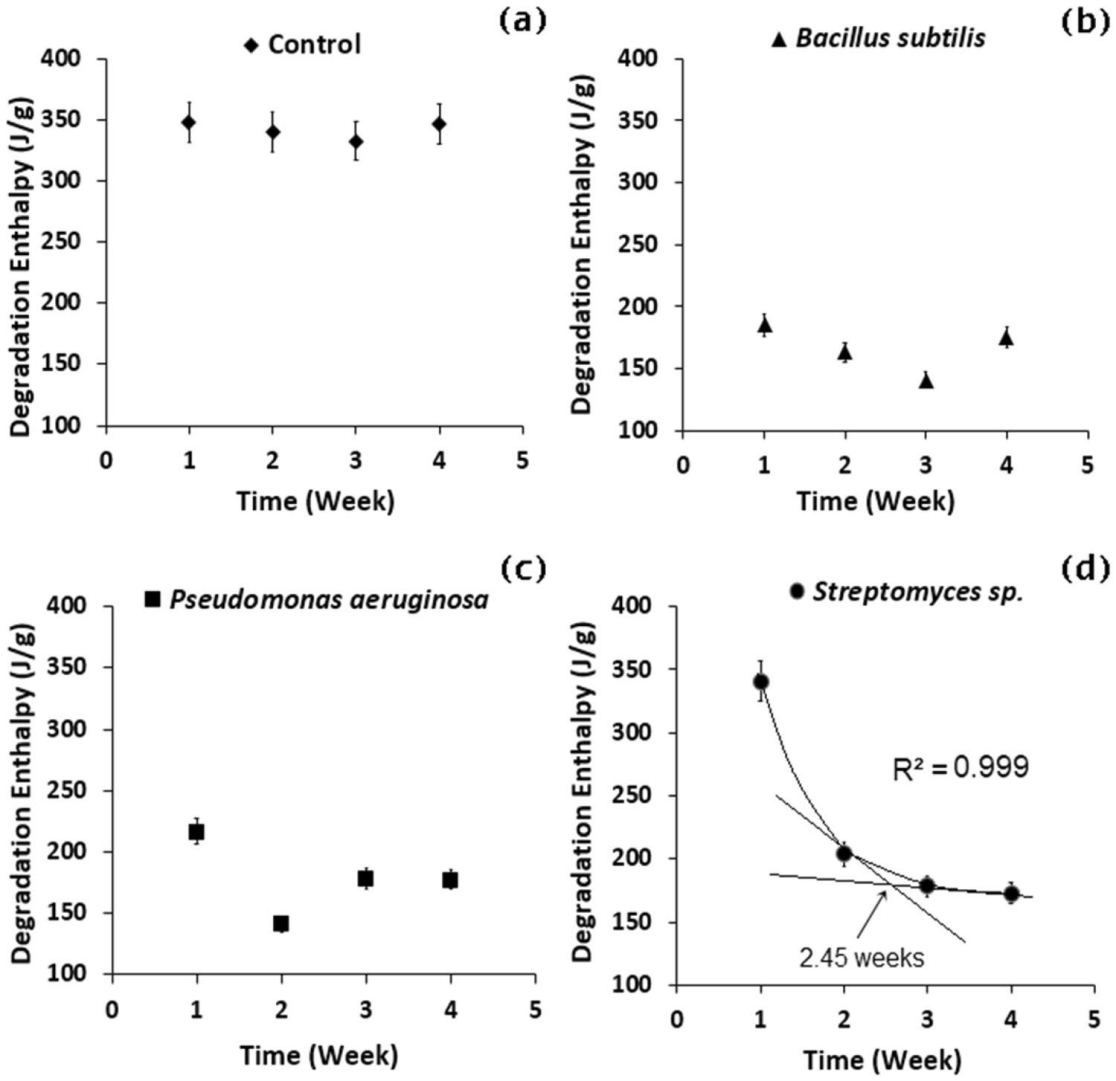
Degradation enthalpy of v-SBR modified by different mediums: (**a**) Control, (**b**) *Bacillus subtilis*, (**c**) *Pseudomonas aeruginosa* and (**d**) *Streptomyces* sp. during 4 weeks of contact with the bacterial strains.

**Figure 6:**
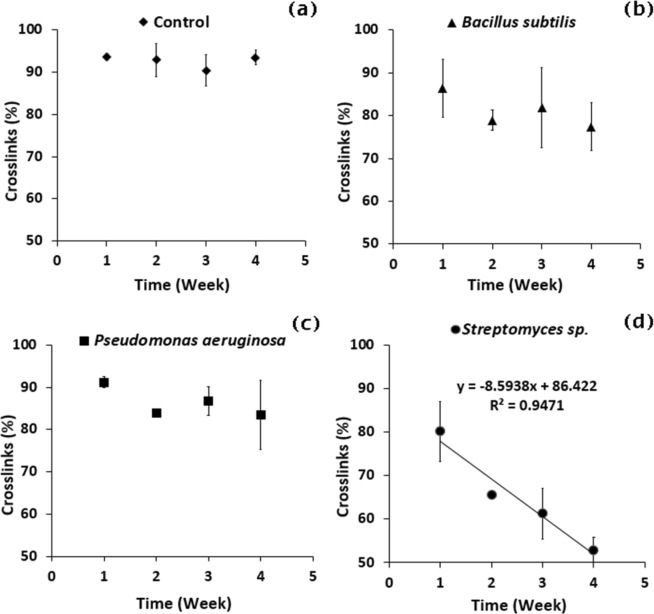
Cross-link degree of v-SBR rubber modified by different mediums: (**a**) Control, (**b**) *Bacillus subtilis*, (**c**) *Pseudomonas aeruginosa* and (**d**) *Streptomyces* sp. during 4 weeks of contact with the bacterial strains.

Figure 5 shows the degradation enthalpy change as a function of time estimated from DSC data, where a descending degradation enthalpy rate was observed by increasing the contact time with all three bacteria. The cross-linked degree has been decreased (see Fig. 6) indicating a scission of cross-linked chains network. The less the crosslinks present in SBR, the less will be the existing bonds in the system, consequently, the lower will be the necessary heat to beak them; i.e. lower degradation enthalpy. v-SBR rubber registered 346.66, 175.27, 177.56 and 172.91 J/g of enthalpy of the main degradation event between the onset and offset temperatures of each sample after contact of 4 weeks with the Control, *B*. *subtilis*, *P*. *aeruginosa*, *and Streptomyces* sp. mediums, respectively. The bacterial strains caused a regressive effect on the degradation enthalpy in v-SBR samples, *B*. *subtilis*, *P*. *aeruginosa*, *and Streptomyces* sp., after 4 weeks when compared to one-week-effect by 23.93, 34.77 and 49.23%, respectively. The optimum contact time of *Streptomyces* sp. With the rubber equals to 2.45 weeks according to the extrapolation proceedings, similar to the one described by ISO 11358, applied to the exponential regression model with R^2^ = 0.999. (see Fig. 5-d).

### Crosslinking degree

Figure 6 shows the cross-link change as a function of time, where a descending rate of residual crosslinking was observed by increasing the contact time with all three bacteria. Decreasing the crosslinking degree indicates the bio-devulcanization/bio-desulfurization and a biodegradative activity of the bacteria on the vulcanized rubber. The v-SBR rubber kept 93.44, 77.42, 83.46 and 52.90% of crosslinking after contact of 4 weeks with the Control, *B*. *subtilis*, *P*. *aeruginosa*, and *Streptomyces* sp. mediums, respectively. The bacterial strains evoked a loss in v-SBR crosslinks by 17.15, 10.68 and 43.39% if compared to Control samples for *B*. *subtilis*, *P*. *aeruginosa*, *and Streptomyces* sp., respectively. The results highlight *Streptomyces* sp. strains for the remarkable biodegradative effect if compared to *B*. *subtilis* or *P*. *aeruginosa*. A linear regression modeling was applied in case of Streptomyces (see Fig. 6-d), where the corresponding correlation coefficient (R^2^) registered 0.95 and the angular coefficient (a) registered – 8.59 (%/week) indicating a remarkable regression rate if compared to Control samples.

### Mass balance of carbon and sulfur

**Figure 7:**
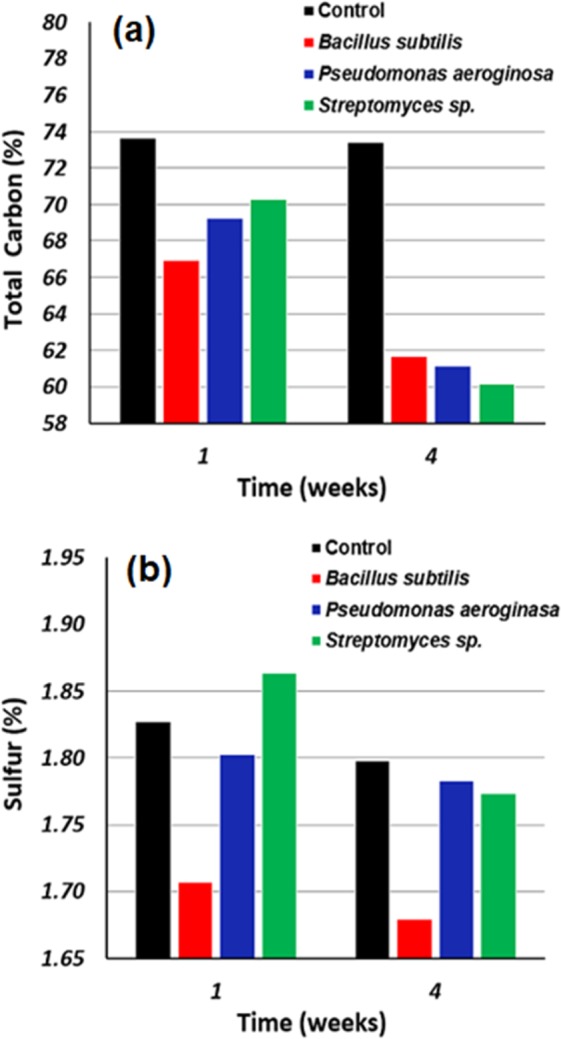
(**a**) Total carbon mass balance and (**b**) sulfur mass balance of the v-SBR samples after one and four weeks of contact with different mediums: Control, *Bacillus subtilis*, *Pseudomonas aeruginosa* and *Streptomyces* sp.

The conversion of the carbons of v-SBR into CO_2_ and the sulfur used in the vulcanization process into SO_2_ was accompanied by a series of carbon and sulfur element analyzing processes after the period of one and four weeks of the treatment. The results showed that the control sample has no significant loss in carbon mass during the test period, while the v-SBR samples that were in contact with the bacteria presented lower total carbon and sulfur percentage by time. This result indicates the biodegradative and devulcanization effect caused by the three bacteria (see Fig. 7). Table 4 in the SI file presents the percentage of the total carbon and sulfur in v-SBR studied samples. The samples in contact with the bacterial strains registered a carbon loss by 9.15, 5.97 and 4.55% after one week and 16.09, 16.79 and 18.13% after four weeks for *B*. *subtilis*, *P*. *aeruginosa*, and *Streptomyces* sp. mediums, respectively, if compared to the control samples. All the three bacteria demonstrated a biodegradative effect on the v-SBR as the carbon was consumed as an energy source during 4 weeks of the essay. Besides that, the bacteria could devulcanize the crosslinked rubber in different scales, highlighting the *Streptomyces* sp. strain by the higher effect corroborating the crosslinking degree and enthalpy results.

The EDS technique has its limitations regarding the capacity of offering precise quantitative element concentrations, but the results are promising and corroborate the crosslinking and the degradation enthalpy results (see Figs. 5 and 6), indicating the bio-devulcanization phenomenon of v-SBR.

Although the literature reports that biodegradation for *Bacillus sp*. S-10 was detected in several strains in the period of 4 to 6 weeks^60^ and for *Streptomyces coelicolor* in 10 to 12 weeks^61^, it can be verified that this study with the strain *Streptomyces* sp. presented the best performance evidence based on Figs. 5 and 6 and Table 4 in the SI file. Perhaps, if the experiment were done for more than 10 weeks, the level of degradation of the rubber could be higher.

The mechanism of biodegradation is not yet fully elucidated, although some hypotheses are presented for actinobacteria, among them the excretion of degrading enzymes. Yikimis and Steinbüchel (2012) reported the expression Latex clearing protein (Lcp), a protein complex responsible for clear zone formation by poly (cis-1,4-isoprene) cleavage in many actinomycetes, such as *Streptomyces* sp. strain K30, *Streptomyces lividans*, and *Saccharopolyspora erythraea*^62^. Linos and Steinbüchel (2005) working in a *Gordonia* consortium culture, have reported that *Pseudomonas* totally lost its ability to grow on rubber after numerous transfers of nutrient media^63^. This could explain the intermediate performance of *Pseudomonas*, compared to the other two strains of this study, since the cultures used as inoculum are being maintained in traditional organic culture media.

Therefore, as *Streptomyces* sp. presented the most effective biodegradation activity on vulcanized rubbers, as shown in the current paper, one can estimate the overall production of a 30 m^3^ bioreactor rich of this microorganism. As per 50 mL of bacteria strain plus the corresponding nutritive medium were sufficient to cause biodegradation of 3.75 g of powdered v-SBR, thus a bioreactor with a volume equals to 30 m^3^ can produce an average of 2.25 tons of devulcanized rubber in one month. A combination of microorganisms like *Streptomyces* sp. and organisms like Tenebrio Molitor Linnaeus larvae can represent a successful biological alliance to devulcanize discarded rubber even faster.

## Conclusions

This paper focuses on comparing the biodegradation activity by *B*. *subtilis*, *P*. *aeruginosa*, and *Streptomyces* sp., separately, on vulcanized SBR-rubber during 4 weeks. From the findings, one can clearly notice that chemical surface modifications occur in v-SBR rubber samples by all the three bacteria through degrading and decomposing carbon and sulfur chemical bonds in the rubber chain system. The biodegradation evoked a bio-devulcanization or bio-desulphurization of the vulcanized rubber polymeric chains. The results are promising and highlighting *Streptomyces* sp. strain as the most effective one in terms of biodegradation and bio-devulcanization. Hopefully, sulfur degrading bacteria can serve as a potential alternative and natural mean of degrading vulcanized rubber residues, which are known to have a very slow degrading rate by default.

## Supplementary information

Supplementary Tables
