# Supplementary material for: Biodegradation of Vulcanized SBR: A Comparison between Bacillus subtilis, Pseudomonas aeruginosa and Streptomyces sp
Source: Sci Rep. 2019 Dec 17;9:19304. doi: 10.1038/s41598-019-55530-y (PMC6917721; doi:10.1038/s41598-019-55530-y)
Supplement: Supplementary file 1 — Supplementary Tables [file 41598_2019_55530_MOESM1_ESM.pdf]

SUPPLEMENTARY INFORMATION (SI)

Biodegradation of Vulcanized SBR: A Comparison between *Bacillus subtilis, Pseudomonas aeruginosa and Streptomyces* sp.

Mostafa G. Aboelkheir[1]; Priscilla B. Bedor[2]; Selma G. Leite[2]; Kaushik Pal[3]; Romildo D. Toledo[5]; Fernando G. Souza Jr[4,5]*

[1.] Programa de Engenharia Civil, Universidade São Judas Tadeu, Rua Taquari, 549, São Paulo, Brasil

[2.] Escola de Química, Centro de Tecnologia - Cidade Universitária, Av. Horacio Macedo, 2030, Bloco E. Universidade Federal de Rio de Janeiro, Brasil, Zip code 21941-909

[3.] Bharath University, BIHER Research Park, Selaiyur, Chennai 600073, Tamil Nadu, India

[4.] Instituto de Macromoléculas, Centro de Tecnologia - Cidade Universitária, Av. Horacio Macedo, 2030, Bloco J. Universidade Federal de Rio de Janeiro, Brasil, Zip code 21941-909

[5.] Programa de Engenharia Civil, COPPE, Centro de Tecnologia-Cidade Universitária, Av. Horacio Macedo, 2030, Bloco I. Universidade Federal de Rio de Janeiro, Brasil, Zip code 21941-914

e-mail: fgsj@ufrj.br

3 pages and 4 tables

Supplementary Table 1 EDS Index of carbon and sulfur elemental analysis presents in v-SBR before and after the contact with three different types of bacteria.

Sample	Element	Weight %	Atomic %	Compound %	Sulfur/Carbon Ratio (%)
Control	C	24.65	31.54	90.33	1.94
	S	0.48	0.23	1.19	
Bacillus subtilis	C	24.86	31.82	91.10	0.47
	S	0.12	0.06	0.29	
Pseudomonas aeruginosa	C	25.25	31.93	92.53	0.64
	S	0.16	0.08	0.40	
Streptomyces sp.	C	25.17	31.78	92.21	0.45
	S	0.11	0.05	0.28	

Supplementary Table 2 Index of vibration modes of the FTIR spectra of v-SBR before and after the contact with bacteria.

Characteristic Band Number	Band Assignments
1	C-H stretching bond in CH_2 and CH_3 groups
2	
3	C=C asymmetric and symmetric stretching in the aromatic ring skeleton
4	
5	
6	S=O & SO_2 conjugated stretching
7	Styrene (cis-1,4-unit, 1,2-unit, and trans-1,4-unit)
8	C-H out-of-plane bend
9	ring out-of-plane C-H bending
10	Styrene (trans-1,4-unit)
Extra Band (1735 cm^{-1})	C=O stretching from residual stearic acid

Supplementary Table 3 Thermal decomposition characteristics of v-SBR before and after the contact with the bacteria during 1 and 4 weeks estimated from TGA data.

Sample	$T_{-2\%}$ (C°)	$T_{-5\%}$ (C°)	$T_{-10\%}$ (C°)	$T_{-50\%}$ (C°)	$T_{-85\%}$ (C°)	Inorganic Residue (%)
Control 1	317	352	384	452	403	6.53
Control 4	320	353	384	453	614	11.94
Bacillus subtilis 1	313	355	383	452	398	4.12
Bacillus subtilis 4	320	358	384	454	484	6.27
Pseudomonas aeruginosa 1	311	352	381	451	396	5.39
Pseudomonas aeruginosa 4	299	351	382	451	397	6.46
Streptomyces sp.*1*	310	351	386	452	404	4.97
Streptomyces sp. *4*	308	351	381	452	482	5.66

Supplementary Table 4 Carbon and Sulfur Mass Balance of v-SBR converted to CO_2 and SO_2 before and after the contact with the bacteria during 1 and 4 weeks.

Sample	Total Carbon (%)	Sulfur (%)	Carbon loss (%)
Control 1	73.64	1.83	0
Control 4	73.45	1.80	0
Bacillus subtilis 1	66.90	1.71	9.15
Bacillus subtilis 4	61.63	1.68	16.09
Pseudomonas aeruginosa 1	69.24	1.80	5.97
Pseudomonas aeruginosa 4	61.12	1.78	16.79
Streptomyces sp.*1*	70.29	1.86	4.55
Streptomyces sp. *4*	60.13	1.77	18.13
